# Knockdown of DEC2 expression inhibits the proliferation of mesangial cells through suppressed glycolysis and p38 MAPK/Erk pathway in lupus nephritis

**DOI:** 10.1186/s10020-023-00672-z

**Published:** 2023-07-24

**Authors:** Huimeng Qi, Li Xu, Qiang Liu

**Affiliations:** 1 Department of General Practice, Fuyang Hospital of Anhui Medical University, Fuyang, 236000 Anhui Province China; 2 Department of Clinical Laboratory, The First Affiliated Hospital of China Medical University, Shenyang, 110001 Liaoning Province China; 3 Department of Nephrology, Guangdong Second Provincial General Hospital, Guangzhou, Guangdong Province China

**Keywords:** DEC2, Mesangial cells, Lupus nephritis, Glycolysis, Bioenergetics

## Abstract

**Background:**

To elucidate the mechanism by which DEC2 modulates the proliferation of mesangial cells (MCs) in lupus nephritis (LN).

**Methods:**

The 32-week-old female Fcgr2b^−/−^ mice and their serum-treated MCs were used as in vivo and in vitro LN model. MCs knocked down of DEC2 and overexpressed with DEC2 were also established. The expression of DEC2 was measured in the kidneys of Fcgr2b^−/−^ mice and LN serum-treated MCs using RT-qPCR and Western blot. MCs proliferation was detected by 5-ethynyl-2′-deoxyuridine (EdU) labeling assay and PCNA expression using immunofluorescence. The glucose metabolism was evaluated in LN serum-treated MCs, and the levels of lactate production, glucose consumption, ATP production and mitochondrial membrane potential were assayed. The glycolysis and mitochondrial respiration function of the MCs were measured using the Extracellular Flux Analyzer. The extracellular acidification rate (ECAR) and oxygen consumption rate (OCR) were dynamically monitored and multiple important bioenergetic parameters can be calculated. The expression of Toll like receptor 4 (TLR4) and glucose transporter 1 (GLUT1) were detected in the MCs. Multiple signaling proteins were screened.

**Results:**

DEC2 was found overexpressed in the kidney of Fcgr2b^−/−^ LN mice. Knockdown of DEC2 inhibited LN serum-induced MCs proliferation. DEC2 was associated with the glucose metabolism in LN serum-treated MCs. DEC2 regulated glycolysis in LN serum-treated MCs. DEC2 was associated with mitochondrial fitness in LN serum treated MCs. DEC2 activated MCs glycolysis through TLR4 and glucose transporter 1 (GLUT1) regulation. DEC2 regulated MCs proliferation through two signaling pathways including dependent and independent of glycolysis, which located in the downstream of TLR4 signaling.

**Conclusion:**

Knockdown of DEC2 expression inhibits the proliferation of MCs through suppressed glycolysis and p38 MAPK/ERK pathway in LN.

## Background

Lupus nephritis (LN), a refractory glomerulonephritis found in the patients with systemic lupus erythematosus, commonly leads to chronic renal failure (Clarke 2021). An important characteristic of LN is the deposition of abundant antigen–antibody complex in the glomeruli, which induces the secretion of pro-inflammatory factors from the activated renal resident cells (Yung and Chan 2017). The immune cells are attracted by inflammatory factors and promotes an inflammatory cascade to influence downstream cell function (Nowling 2022). Multiple stubborn symptoms occurs in LN, such as hyperplastic glomerular capillary and mesangial expansion (Qi et al. 2020).

Mesangial cells (MCs), locate among the capillary loops in the glomeruli, provide structural support and maintain the glomerular function in kidneys (Zhao et al. 2021). As the renal resident cells, MCs are commonly involved in variety of kidney diseases including LN when the glomerular environment is influenced by multiple pathological stimuli. In LN, MCs are early responders to deposited immune complex and activated to induce inflammatory reaction (Nowling 2022). The functional change of MCs, especially the excessive proliferation in LN, can injure glomerular capillaries and decrease the glomerular filtration rate (Tung et al. 2018; Ebefors and Nyström 2017). Thus, MCs are key cell type to promote the progression of LN (Kwok and Tsokos 2018).

DEC2, also known as BHLHE41, is a transcription factor showing inhibitory effects on E-box-mediated transcription (Kato et al. 2014), thereby participating in the fine-tuning of the circadian clock (Honma et al. 2002). Imaizumi et al. (2011) reported that DEC2 constituted negative feedback loop in IFN-β/RIG-I/CCL5 axis, playing inflammation-inhibitory role in human MCs. We previously found that downregulation of DEC2 and the Toll like receptor 4 (TLR4) signaling inactivation inhibited the proliferation of MCs (Qi et al. 2020). However, the bioenergetics that support this process remain poorly elucidated. Except for the regulative effects of pro-proliferative signaling, cell proliferation is also potentially promoted by the modulation of cellular bioenergetics (Beck et al. 2015). In this study, the roles of glycolysis and mitochondrial function in the proliferation of MCs were evaluated and related signaling pathways were also elucidated.

## Materials and methods

### Mice and the collection of LN serum

All the animal experiments in this study were approved by the Ethical Committee of Anhui Medical University. The LN model mice used in this study are 32-week-old female C57BL/6 mice (n = 30) with specific Fcgr2b gene deficiency (Fcgr2b^−/−^ mice, Strain No. 002848), which were from Jackson Laboratory (Bar Harbor, ME). The Fcgr2b^−/−^ mice can spontaneously develop a lupus-like pathological changes including LN after 32 weeks old (Qi et al. 2020; Pisitkun et al. 2012). Age-matched female C57BL/6 mice (n = 30), purchased from Shanghai SLAC Laboratory Animal Co., Ltd. (Shanghai, China), served as controls. To evaluate the effect of DEC2 on the development of LN, 22 week-old female C57BL/6 mice (n = 12) were randomly divided into DEC2 knockdown [Sh-DEC2, short hairpin RNA (shRNA) targeting DEC2] group and its negative control group (Sh-NC). The vector genome of DEC2-shRNA-AAV or NC-shRNA-AAV in 50 µL physiological saline was administered into the renal cortex of the mice, which were raised for 10 weeks until sacrifice. To determine the level of proteinuria, the urines of the mice in each group were collected and the ratio of albumin to creatinine (ACR) was assayed. To simulate the LN circumstances, the blood was collected from the 32 week-old female C57BL/6 mice, and the LN serum was collected after centrifugation. Serum isolated from age-matched female C57BL/6 mice served as controls.

### Periodic acid-Schiff (PAS) staining

The mice were sacrificed by excessive dose of the anesthesia with pentobarbital sodium. The kidney tissue samples were collected and fixed in formalin. The specimens were embedded in paraffin and sectioned at 4 μm thickness, deparaffinized in xylene and hydrated using ethanol to distilled water. Each slide was incubated with 100 µL periodic acid solution for 10 min and 100 µL Schiff reagent (Beyotime Biotechnology, Shanghai, China) for 1 h in a dark wet box. The slides were then rinsed in distilled water for 5 min and stained with 100 µL hematoxylin solution for 30 s, and rinsed in distilled water. The samples were differentiated by hydrochloric acid–ethanol mixtures, rinsed in distilled water and sealed with neutral balsam.

### Immunohistochemistry

The renal slides (4 μm thickness) were deparaffinized to distilled water and incubated with citrate buffer for antigen microwave retrieval. The slides were then washed with phosphate-buffered saline (PBS) and blocked using PBS containing 5% bovine serum albumin (BSA). The slides were incubated with primary antibodies including anti-DEC2 (1:200) and anti-PCNA (1:200, both from ThermoFisher Scientific, Waltham, MA, USA) overnight in a wet box at 4 °C. The slides were then incubated with corresponding HRP-conjugated secondary antibodies (ThermoFisher Scientific) at room temperature for 1 h and followed by diaminobenzidine and hematoxylin staining.

### Cell culture

The MCs used in this study were from a mouse kidney mesangial cell line (SV40 MES-13, RRID: CVCL_5368), purchased from Shanghai Institute of Biochemistry and Cell Biology, Chinese Academy of Sciences (Shanghai, China). The MCs were cultured in the Dulbecco’s modified Eagle medium (DMEM, Gibco, Grand Island, NY, USA) supplemented with 10% fetal bovine serum (Gibco). Different treatments of the cells were separately represented as follows: (1) The MCs were serum-starved overnight and exposed to LN serum (5% in DMEM) or control serum (5% in DMEM) for 24 h. The serum was detected for the endotoxin in advance and only serum with undetectable level of endotoxin could be used in this study. (2) The MCs were transfected with shRNA targeting DEC2 (Sh-DEC2) or negative control (Sh-NC). (3) The MCs were transfected with lentiviral vector overexpressing DEC2 (Lv-DEC2) or negative control (Lv-NC). (4) The MCs were treated with multiple inhibitors of signaling pathways, including TLR4-specific antagonist chloroquine phosphate (CP, MedChemExpress, Monmouth Junction, NJ, USA), PI3K inhibitor LY294002 (Beyotime Biotechnology), Akt inhibitor AZD5363 (Beyotime Biotechnology), NF-κB inhibitor BAY 11-7082 (Beyotime Biotechnology), p38 MAPK inhibitor SB203580 (Beyotime Biotechnology) and Erk inhibitor FR 180204 according to the manufacturer’s instructions.

### Real time quantitative polymerase chain reaction (RT-qPCR)

The total RNAs were extracted from the MCs with TRIzol regent. The RNA precipitates were quantified with a NanoQuant spectrophotometer (Tecan, Vienna, Austria). A High-Capacity cDNA Reverse Transcription Kit (ThermoFisher Scientific) was used to reversely transcribe the RNA into cDNA. RT-qPCR was performed with DEC2-specific primers (forward, 5′-AACATGGACGAAGGAATCCCTC, and reverse, 5′-TAAGGCTGTTAGCGCTTTCAAG) and using a Rotor-Gene SYBR Green PCR Kit (Qiagen GmbH, Hilden, Germany). GAPDH (forward, 5′-CATCACTGCCACCCAGAAGACTG and reverse, 5′-ATGCCAGTGAGCTTCCCGTTCAG) were used as internal control. Relative RNA expression was determined by the 2^−ΔΔCT^ method.

### Western blot

The proteins were extracted from the MCs using radio immunoprecipitation assay (RIPA) lysis buffer (Beyotime Biotechnology) containing protease and phosphatase inhibitor cocktail (Abcam, Cambridge, MA, USA). Equal amount of cell lysates were separated by sodium dodecylsulfate-polyacrylamide gel electrophoresis (SDS-PAGE) at suitable concentration according to the molecular weight of target protein. The proteins were then transferred to a polyvinylidene difluoride (PVDF) membrane (Millipore, Billerica, MA, USA), which was blocked by 5% BSA and then incubated with primary antibodies overnight in a wet box at 4 °C. Primary antibodies used in this study included anti-DEC2 (PA5-106529, 1:1000, ThermoFisher Scientific), TLR4 (ab13556, 1:1000, Abcam), glucose transporter 1 (GLUT1, ab115730, 1:1000, Abcam), p-p38/p38 (#4631 and #9212, 1:1000, Cell Signaling Technology, Danvers, MA), p-Erk/Erk (#4370 and #4695, 1:1000, Cell Signaling Technology), p-PI3K/PI3K (ab182651 and ab191606, 1:1000, Abcam), p-Akt/Akt (ab131443 and ab179463, 1:1000, 1:5000, Abcam), NF-κB (ab32360, 1:1000, Abcam), GAPDH (ab8245, 1:2000, Abcam) and β-actin (ab8226, 1:2000, Abcam). After incubation with the corresponding secondary antibodies (1:2000, ThermoFisher Scientific), the positive bands were visualized using the BeyoECL Plus chromogenic kit (Beyotime Biotechnology), and analyzed by the Tanon-4200 Gel Imaging System (Tanon, Shanghai, China).

### Immunofluorescence

To determine the proliferation level of MCs, we performed the 5-ethynyl-2′-deoxyuridine (EdU) labeling assay and observed the expression level of PCNA using immunofluorescence. The incorporation of EdU was observed with a Zeiss Axio Observer Z1 microscope (Carl Zeiss, Thornwood, NY, USA) after the MCs were incubated with EdU solution (Beyotime Biotechnology) according to the manufacturer’s instructions. For the staining of PCNA, the MCs were fixed in 4% paraformaldehyde solution and permeabilized with 0.5% Triton X-100 for 15 min. After blocked by 5% BSA, the cells were incubated with anti-PCNA antibody (#13-3900, 1:100, ThermoFisher Scientific) and corresponding secondary antibody (ThermoFisher Scientific) and 4′,6-diamidino-2-phenylindole (DAPI, Beyotime Biotechnology). The samples were observed with a Carl Zeiss microscope.

### Lactate content assay

The MCs were treated with different stimuli or inhibitors in DMEM for 24 h. The concentration lactate was determined by using l-Lactate Assay Kit (Eton Biosciences, San Diego, CA, USA) according to the manufacturer’s instruction. The results were reported and analyzed with a Synergy4 microplate reader (BioTek Instruments, Winooski, VT, USA).

### Glucose consumption assay

The consumption of glucose by MCs was measured with 2-NBDG, a fluorescent glucose uptake probe (ThermoFisher Scientific). The cells were seeded into a 96-well plate and treated with LN serum or control serum for 24 h. Then the MCs were rinsed with Hanks balanced salt solution (HBSS, Gibco, ThermoFisher Scientific, Waltham, MA, USA) and incubated with 100 μM 2-NBDG at 37 °C for 30 min. The results were reported and analyzed with a Synergy4 microplate reader (BioTek Instruments).

### Mitochondrial membrane potential assay

The Mitochondrial Membrane Potential Assay Kit with JC-1 (Beyotime Biotechnology) was used to detect the mitochondrial membrane potential of MCs treated with or without LN serum. After treatments for 0, 1, 2, 4 and 6 h, the cells were rinsed with HBSS and incubated with 10 μM JC-1 at 37 °C for 30 min. The fluorescent intensity of the cells was detected according to the manufacturer’s instructions.

### ATP level assay

The ATP Assay Kit (Beyotime Biotechnology) was used to detect the ATP level of MCs treated with or without LN serum. Briefly, the MCs were treated with lysis buffer to release the cellular ATP. Then the lysate was centrifuged at 12,000*g* for 5 min at 4 ºC. The supernatant was mixed well with the ATP detecting reagent (containing firefly luciferase and luciferin) and measured on a Synergy4 microplate reader (BioTek Instruments), which has a standard luminescence setting.

### Glycolysis stress test

The glycolytic function of MCs in each group was detected using a Seahorse XF Glycolysis Stress Test Kit (Agilent Technologies, Santa Clara, CA, USA). The change of glycolysis in treated cells was monitored in real-time by directly probe the values of extracellular acidification rate (ECAR) using the Extracellular Flux Analyzer (XFe24, Agilent Technologies). The MCs were seeded in a XFe24 culture plate and treated with different stimuli or inhibitors in DMEM for 24 h. The medium was then replaced by the Seahorse XF assay medium according to the manufacturer's instruction until assay. The ECAR was dynamically monitored along with the sequential addition of glucose (10 mM), oligomycin (1 µM), and 2-deoxyglucose (2-DG, 50 mM). Several important parameters including glycolysis, glycolytic capacity and glycolytic reserve can be calculated (https://www.agilent.com).

### Mitochondrial stress test

The mitochondrial function of the MCs in each group was detected using a Seahorse XF Cell Mito Stress Test Kit (Agilent Technologies). The change of mitochondrial energy pathway in treated cells was monitored in real-time by directly probe the values of oxygen consumption rate (OCR) using the Extracellular Flux Analyzer (XFe24, Agilent Technologies). The MCs were seeded in a XFe24 culture plate and treated with different stimuli or inhibitors in DMEM for 24 h. The medium was then replaced by the Seahorse XF assay medium according to the manufacturer's instruction until assay. The OCR was dynamically monitored along with the sequential addition of oligomycin (100 µM), FCCP (100 µM), and rotenone/antimycin A (50 µM). Several important parameters including basal respiration, ATP production-related respiration, maximal respiration, spare capacity and proton leak-related respiration can be calculated (https://www.agilent.com).

### Statistical analysis

Statistical analyses were performed with SPSS 20.0 software (IBM, Armonk, NY, USA). The data are shown as the mean ± standard deviation. The data were subjected to t-test or one-way ANOVA with a post hoc multiple comparison. A *P*-value < 0.05 was considered statistically significant.

## Results

### DEC2 is overexpressed in the kidney of Fcgr2b^−/−^ LN mice

**Fig. 1:**
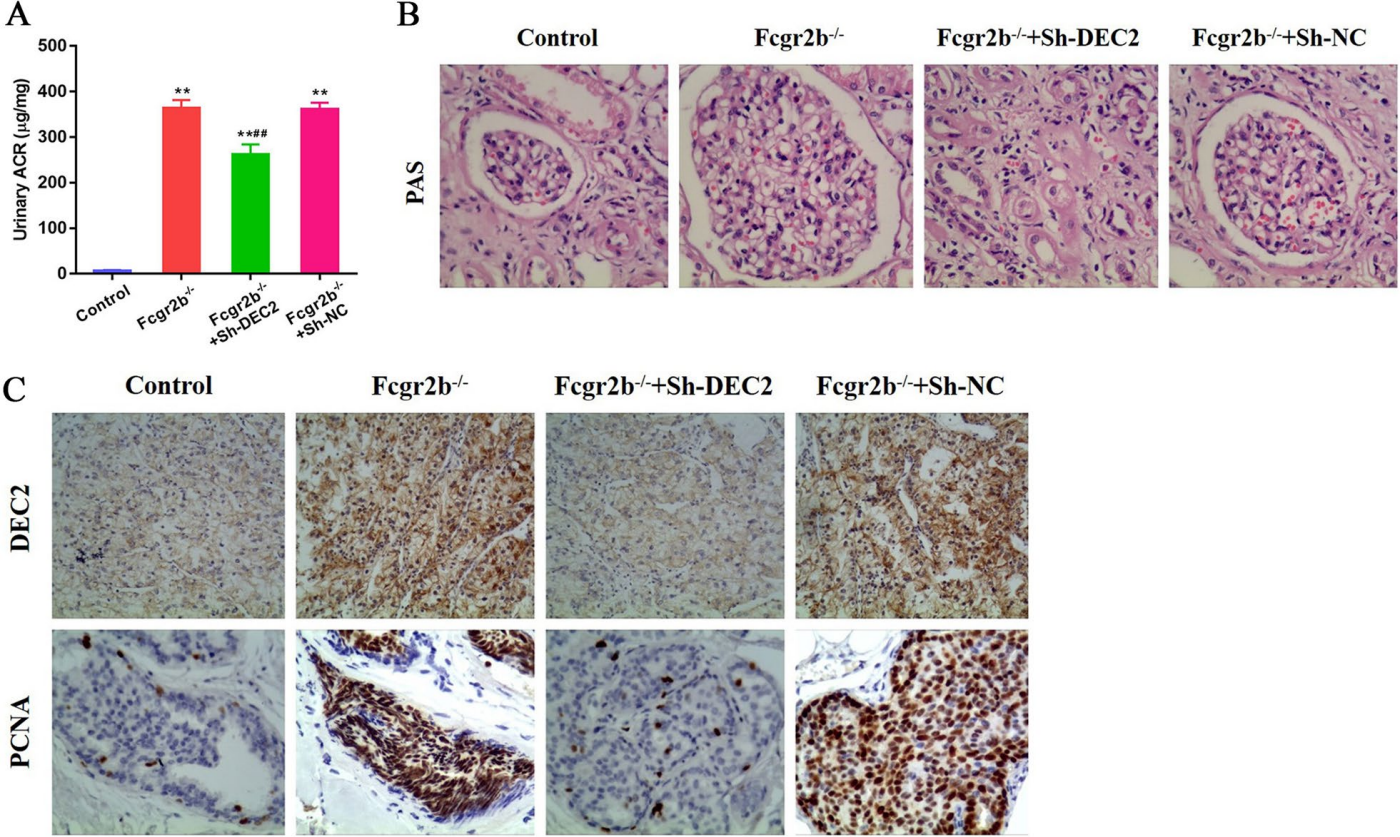
DEC2 is overexpressed in the kidney of Fcgr2b^−/−^ LN mice. **A** Increased urinary ACR, a marker for proteinuria, was found in Fcgr2b^−/−^ mice. Knockdown of DEC2 in kidney tissue partly reversed the proteinuria in mice. **B** PAS staining showed MCs proliferation and capillary hyperplasia in Fcgr2b^−/−^ mice, and decreased glomerular injury was found in Fcgr2b^−/−^ mice with DEC2 Knockdown. **C** IHC staining proved that DEC2 was overexpressed in the kidney of Fcgr2b^−/−^ LN mice and a high expression of proliferative marker PCNA found in kidney tissue of Fcgr2b^−/−^ mice. **P < 0.01 vs. control; ^##^*P* < 0.01 vs. Fcgr2b^−/−^ + Sh-NC

Previous evidence indicated that the female Fcgr2b^−/−^ mice spontaneously developed LN-like symptoms from 32-week old (Qi et al. 2020). The LN proteinuria was proved by significantly increased urinary ACR in Fcgr2b^−/−^ mice compared with that in controls (P < 0.01). Using orthotopic injection method, the kidneys of the Fcgr2b^−/−^ mice were administered DEC2-shRNA-AAV (Sh-DEC2) or NC-shRNA-AAV (Sh-NC). The level of urinary ACR in Fcgr2b^−/−^ mice with Sh-DEC2 was significantly decreased than that in Fcgr2b^−/−^ mice with Sh-NC (P < 0.01), suggesting that knockdown of DEC2 alleviated the development of LN proteinuria in Fcgr2b^−/−^ mice (Fig. 1A). MCs proliferation and mesangial expansion were found in Fcgr2b^−/−^ mice and glomerular capillary hyperplasia and glycogen deposition were also observed using PAS staining. Decreased glomerular injury was found in Fcgr2b^−/−^ mice with Sh-DEC2 other than Sh-NC (Fig. 1B). The results of IHC indicated that DEC2 is overexpressed in the kidney of Fcgr2b^−/−^ LN mice. The expression of DEC2 was effectively suppressed following the Fcgr2b^−/−^ mice received Sh-DEC2 other than Sh-NC. The expression of PCNA, mainly in cell nuclei, was significantly higher in Fcgr2b^−/−^ mice compared with that in controls. Decreased expression of PCNA was found in the glomerulus of the Fcgr2b^−/−^ mice received Sh-DEC2 other than Sh-NC, suggesting that knockdown of DEC2 inhibited cell proliferation in the LN glomerulus (Fig. 1C).

### Knockdown of DEC2 inhibits LN serum-induced MCs proliferation

**Fig. 2:**
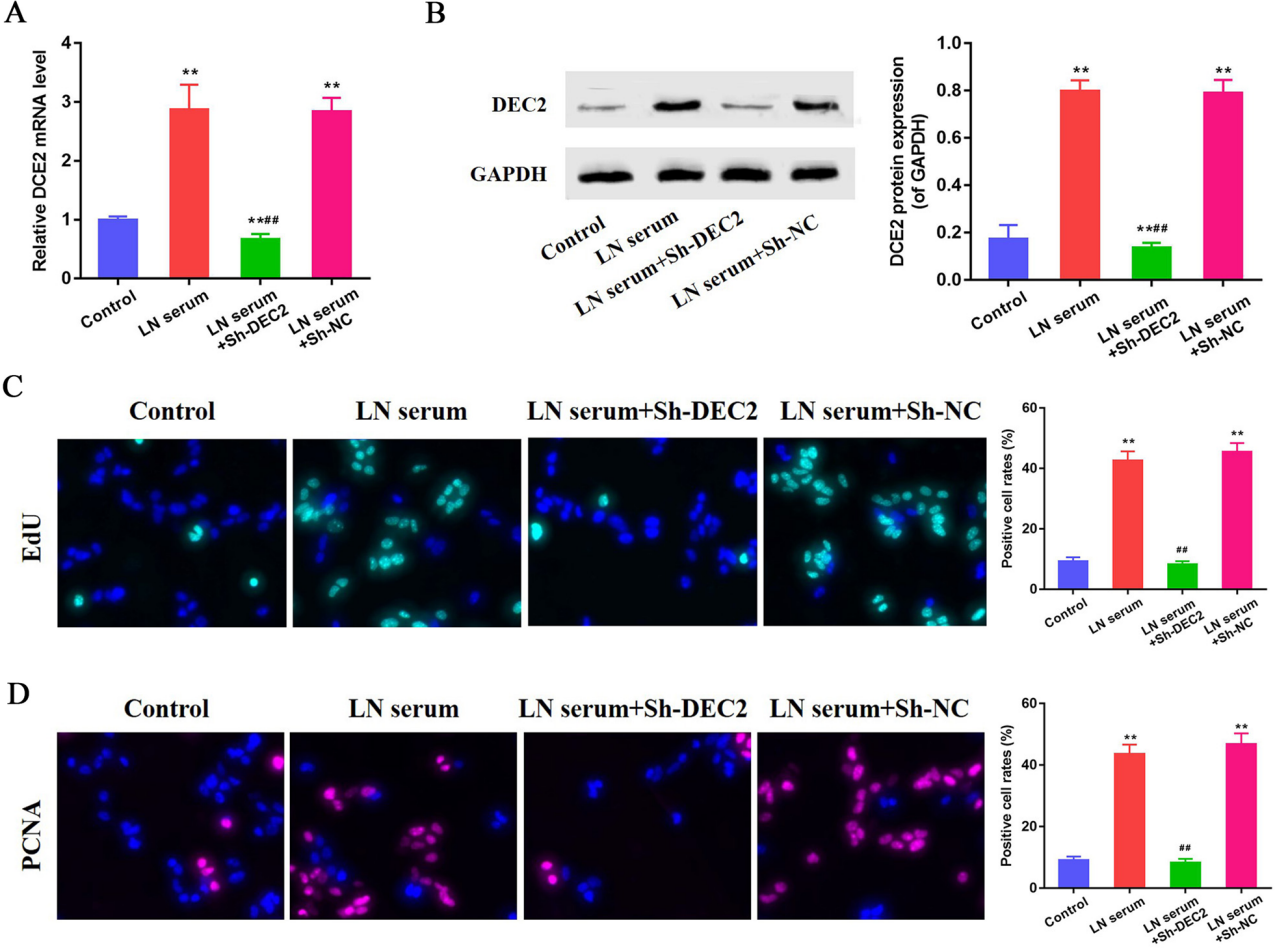
Knockdown of DEC2 inhibits LN serum-induced MCs proliferation. **A**, **B** LN serum induced a higher expressions of DEC2 mRNA and protein in MCs. The MCs transfected with Sh-DEC2 showed significantly decreased levels of DEC2 mRNA and protein although treated with LN serum. **C** EdU staining indicated that increased proliferation was found after the MCs treated with LN serum, and the cells transfected with Sh-DEC2 reversed this effect. **D** This result was further confirmed by the expression of PCNA in LN serum-treated MCs with or without transfected with Sh-DEC2. **P < 0.01 vs. control; ^##^*P* < 0.01 vs. LN serum + Sh-NC

The serum from 32-week Fcgr2b^−/−^ mice was used to simulate the LN circumstances that the MCs are exposed. The level of DEC2 mRNA was significantly up-regulated after exposed to LN serum compared to that in control cells (P < 0.01). The MCs transfected with DEC2-specific shRNA (Sh-DEC2) showed significantly decreased level of DEC2 mRNA compared with that in MCs transfected with negative control shRNA (Sh-NC, P < 0.01, Fig. 2A). The expression of DEC2 protein was significantly up-regulated after treatment with LN serum compared to that in controls (P < 0.01). The MCs transfected with Sh-DEC2 showed significantly decreased expression of DEC2 protein compared with that in MCs transfected with Sh-NC (P < 0.01, Fig. 2B). The EdU staining was used to evaluate the proliferation of MCs exposed to LN serum. The results indicated that increased proliferation was found after the MCs treated with LN serum, and the cells transfected with Sh-DEC2, other than Sh-NC, reversed this effect (Fig. 2C). These data suggest that DEC2 plays important role in the proliferation of MCs exposed to LN stimulus. This result was further confirmed by the expression of PCNA in LN serum-treated MCs with or without transfected with Sh-DEC2 (Fig. 2D).

### DEC2 is associated with the glucose metabolism in LN serum-treated MCs

**Fig. 3:**
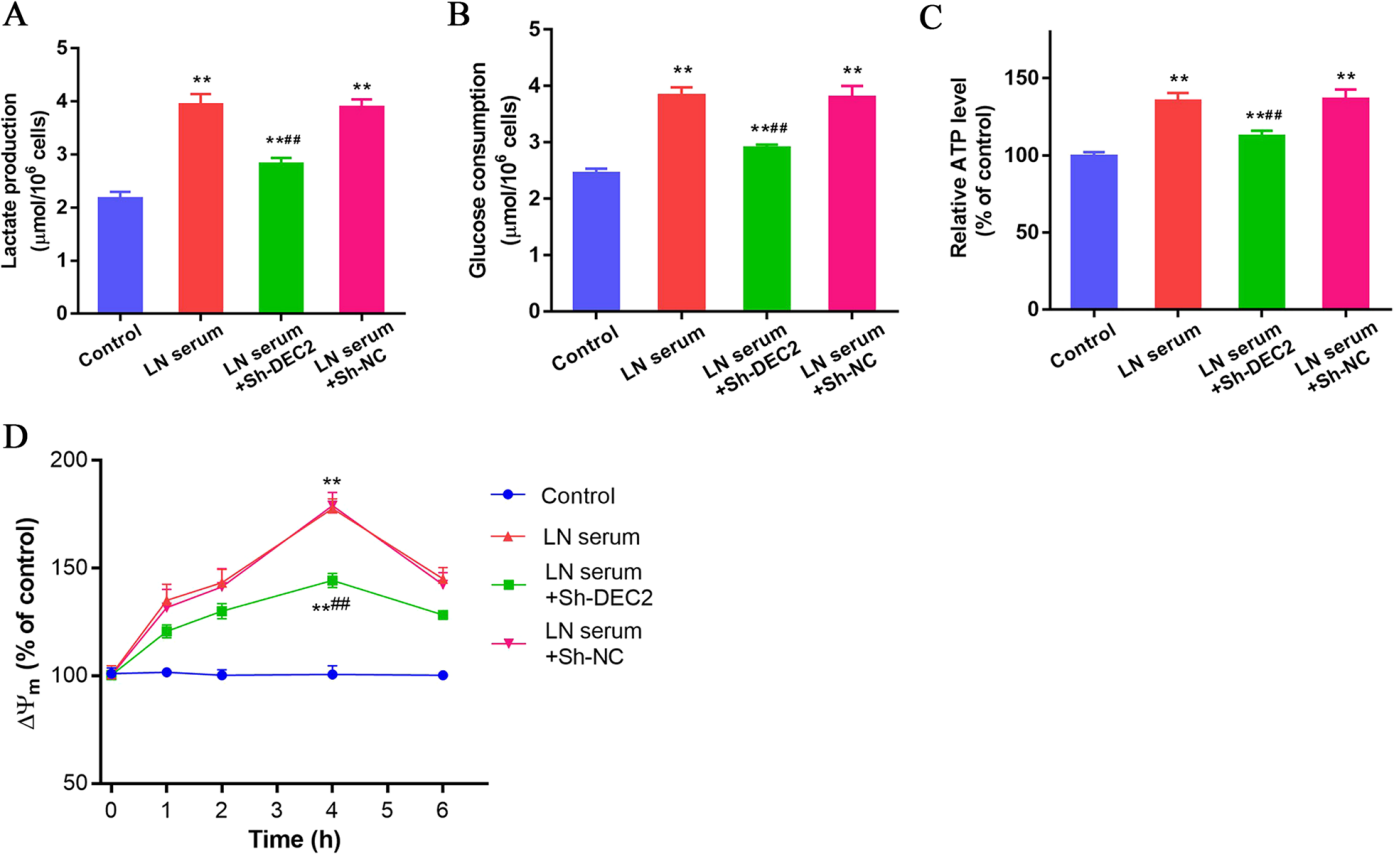
DEC2 is associated with the glucose metabolism in LN serum-treated MCs. **A** LN serum induced a higher lactate production in MCs, and DEC2 knockdown partly reversed this effect in LN serum-treated MCs. **B** LN serum treatment increased the glucose consumption in MCs, and DEC2 knockdown partly reversed this effect in LN serum-treated MCs. **C** LN serum induced a higher ATP production in MCs, and DEC2 knockdown partly reversed this effect in LN serum-treated MCs. **D** The mitochondrial membrane potential increased rapidly in LN serum-treated MCs than that in controls, and significantly decreased in the LN serum-treated cells transfected with Sh-DEC2. **P < 0.01 vs. control; ^##^*P* < 0.01 vs. LN serum + Sh-NC

Cellular bioenergetics are at the foundation of multiple biological activities including cell proliferation (Zhu and Thompson 2019). Lactate production was found significantly increased in LN serum-treated MCs compared with that in control cells (P < 0.01). The level of lactate was decreased in the LN serum-treated cells transfected with Sh-DEC2, other than Sh-NC (P < 0.01, Fig. 3A). We then detected the level of glucose consumption and ATP production to determine whether glucose metabolism is involved in the regulation of DEC2 on the proliferation of MCs. Indeed, the glucose consumption and ATP level were found significantly increased in LN serum-treated MCs compared with those in control cells (P < 0.01). These levels significantly decreased in the LN serum-treated cells transfected with Sh-DEC2 than those in LN serum-treated cells with Sh-NC (P < 0.01, Fig. 3B, C). Change in mitochondrial membrane potential was dynamically assayed in each cell group. The mitochondrial membrane potential increased rapidly in LN serum-treated MCs than that in controls, and significantly decreased in the LN serum-treated cells transfected with Sh-DEC2, other than Sh-NC (both P < 0.01, Fig. 3D).

### DEC2 regulates glycolysis in LN serum-treated MCs

**Fig. 4:**
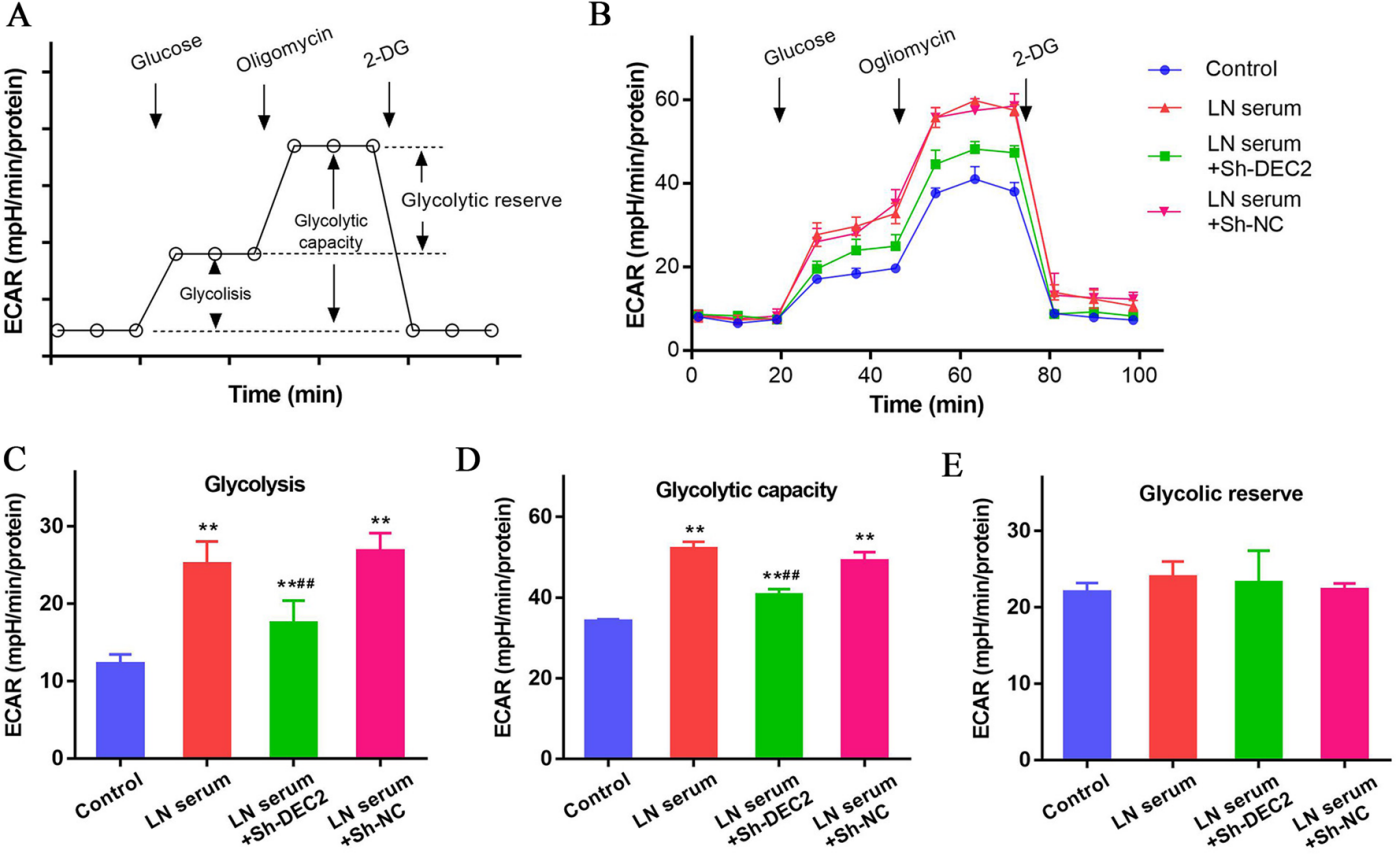
DEC2 regulates glycolysis in LN serum-treated MCs. **A** Diagram for the glycolytic function assay. **B** ECAR of the MCs was dynamically monitored along with the sequential addition of glucose, oligomycin and 2-DG. Multiple parameters including glycolysis, glycolytic capacity and glycolytic reserve can be calculated. **C**, **D** Both glycolysis level and glycolytic capacity significantly increased in the LN serum-treated MCs compared with those in controls, these increases in glycolysis level and glycolytic capacity can be partly reversed after knockdown of DEC2 in the MCs. **E** No statistical differences were found in the glycolytic reserve among groups. **P < 0.01 vs. control; ^##^*P* < 0.01 vs. LN serum + Sh-NC

The schematic diagram of glycolytic function assay is shown in Fig. 4A. ECAR of the MCs was dynamically monitored along with the sequential addition of glucose, oligomycin and 2-DG. Multiple parameters including glycolysis, glycolytic capacity and glycolytic reserve can be calculated according to the formulas provided by the manufacturer (Fig. 4B). The glycolysis level was significantly increased in the LN serum-treated MCs compared with that in controls. Decreased glycolysis level was observed in LN serum-treated MCs transfected with Sh-DEC2, other than Sh-NC (All P < 0.01, Fig. 4C). Similarly, the glycolytic capacity was significantly increased in the LN serum-treated MCs compared with that in controls. Decreased glycolytic capacity was found in LN serum-treated MCs transfected with Sh-DEC2, other than Sh-NC (All P < 0.01, Fig. 4D). However, no statistical differences were found in the glycolytic reserve among groups (Fig. 4E). These data suggest that LN serum activates the glycolysis in MCs and DEC2 regulates glycolysis in LN serum-treated MCs.

### DEC2 is associated with mitochondrial fitness in LN serum treated MCs

**Fig. 5:**
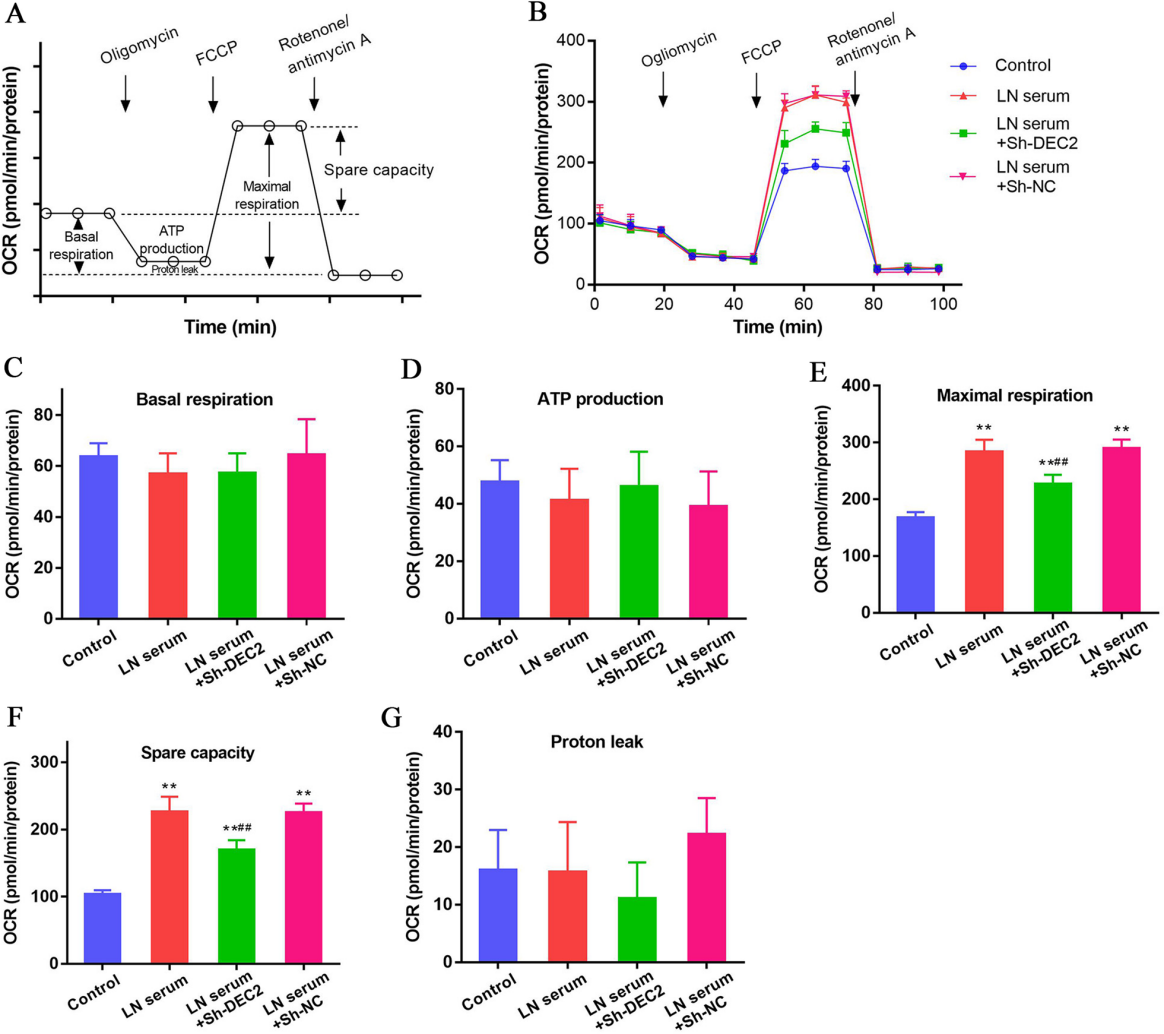
DEC2 is associated with mitochondrial fitness in LN serum treated MCs. **A** Diagram for the mitochondrial respiration function. **B** OCR of the MCs was dynamically monitored along with the sequential addition of oligomycin, FCCP and rotenone/antimycin A. Multiple parameters including basal respiration, ATP production-linked respiration, maximal respiration, spare capacity and proton leak can be calculated. **C**, **D** No statistical differences were found in basal respiration and ATP production-linked respiration among each group. **E**, **F** Increased maximal respiration and spare capacity were found in the LN serum-treated MCs compared with those in controls. Decreased maximal respiration and spare capacity were observed in LN serum-treated MCs transfected with Sh-DEC2, other than Sh-NC. **G** No statistical difference was found in proton leak-linked respiration among each group. **P < 0.01 vs. control; ^##^*P* < 0.01 vs. LN serum + Sh-NC

The schematic diagram of mitochondrial respiration function assay is shown in Fig. 5A. OCR of the MCs was dynamically monitored along with the sequential addition of oligomycin, FCCP and rotenone/antimycin A. Multiple parameters including basal respiration, ATP production-linked respiration, maximal respiration, spare capacity and proton leak can be calculated according to the formulas provided by the manufacturer (Fig. 5B). No statistical differences were found in basal respiration and ATP production-linked respiration among each group (Fig. 5C, D). Increased maximal respiration and spare capacity were found in the LN serum-treated MCs compared with those in controls. Decreased maximal respiration and spare capacity were observed in LN serum-treated MCs transfected with Sh-DEC2, other than Sh-NC (All P < 0.01, Fig. 5E, F). In addition, no statistical difference was found in proton leak-linked respiration among each group (Fig. 5G). These data suggest that DEC2 is associated with mitochondrial fitness in LN serum-treated MCs.

### DEC2 activates MCs glycolysis through TLR4 and glucose transporter 1 (GLUT1) regulation

**Fig. 6:**
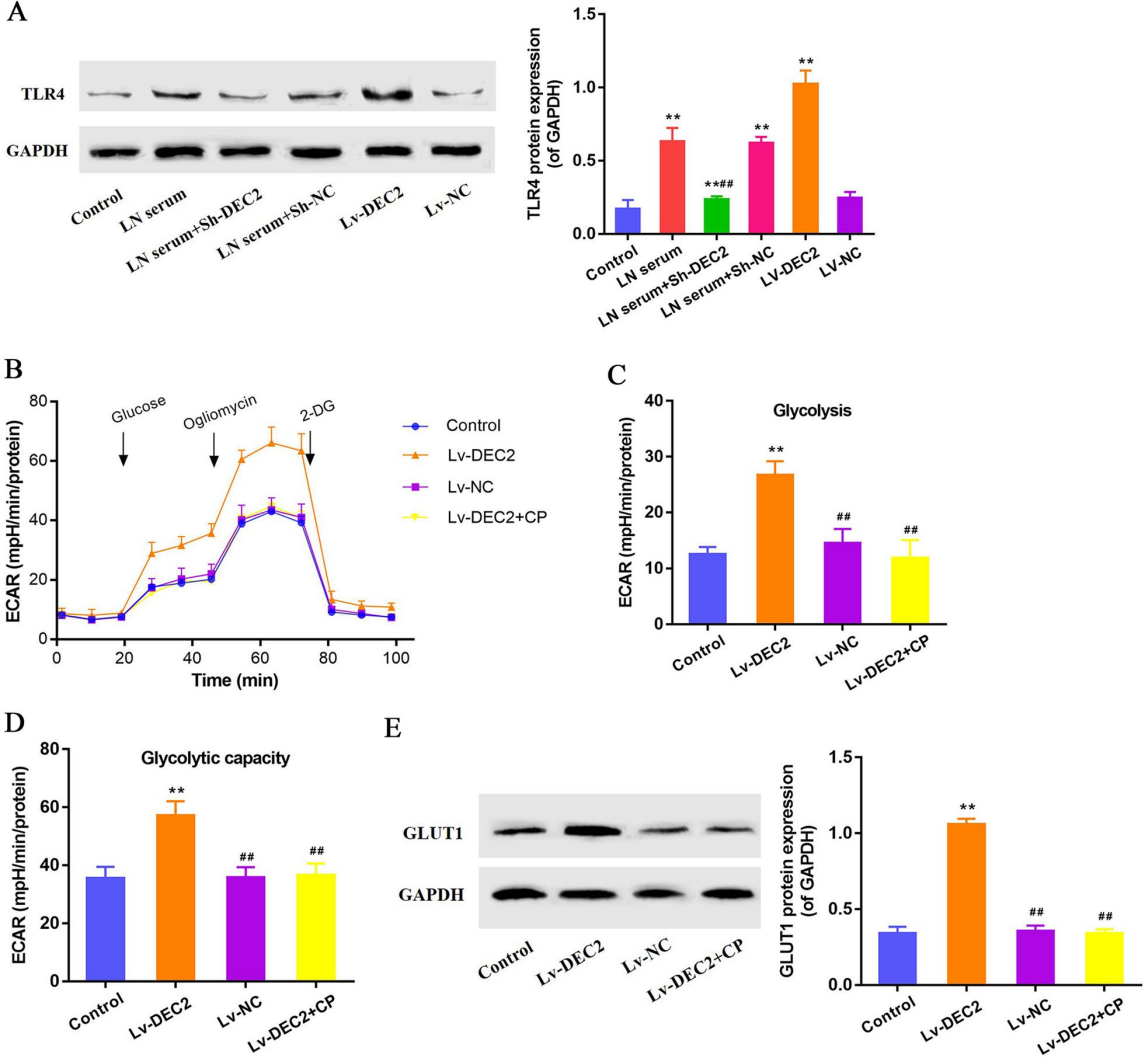
DEC2 activates MCs glycolysis through TLR4 and glucose transporter 1 (GLUT1) regulation. **A** Western blot results showed that LN serum activated TLR4 signaling, and DEC2 knockdown inhibited the LN serum-induced TLR4 activation in MCs. Overexpression of DEC2 could also activate the TLR4 signaling. **B** Increased ECAR value was monitored in MCs overexpressed with DEC2 and TLR4 inhibitor CP suppressed the elevation of ECAR value. **C**, **D** Overexpression of DEC2 significantly activated the glycolysis and glycolytic capacity in MCs, and TLR4 inhibitor CP effectively suppressed the elevation of ECAR values. **E** Overexpression of DEC2 increased the expression of GLUT1 in MCs and CP treatment suppressed the elevation of GLUT1 expression. **P < 0.01 vs. control; ^##^*P* < 0.01 vs. LN serum + Sh-NC and Lv-DEC2

To determine whether DEC2 regulates glycolysis in MCs through TLR4 pathway, we detected the expression of TLR4 in the MCs with or without LN serum treatment and knockdown or overexpression of DEC2. The result of Western blot showed that TLR4 was activated in the LN serum-treated MCs. Decreased expression of TLR4 was found in LN serum-treated MCs transfected with Sh-DEC2, other than Sh-NC. In addition, overexpression of DEC2 (Lv-DEC2) significantly activated the TLR4 pathway other than Lv-NC (Fig. 6A). We then evaluated the role of TLR4 in DEC2-induced MCs glycolysis activation. Increased ECAR value was monitored in MCs overexpressed with DEC2 and TLR4 inhibitor chloroquine phosphate (CP) suppressed the elevation of ECAR value (Fig. 6B). The glycolysis level was significantly increased in MCs overexpressed with DEC2 and CP treatment effectively inhibited DEC2-associated glycolysis activation in MCs (Both P < 0.01, Fig. 6C). Similarly, glycolytic capacity was also significantly increased in MCs overexpressed with DEC2 and CP treatment effectively inhibited DEC2-associated increase in glycolytic capacity (Both P < 0.01, Fig. 6D). Previous report indicated that glucose transporter 1 (GLUT1) could be up-regulated by TLR pathway and promoted glycolysis activation (Akira and Takeda 2004). We detected the expression of GLUT1 in MCs and the Western blot result indicated that Lv-DEC2 increased the expression of GLUT1 in MCs and CP treatment significantly suppressed the elevation of GLUT1 expression (Fig. 6E). These data indicate that DEC2 activates MCs glycolysis through TLR4 and GLUT1 regulation.

### DEC2 regulates MCs proliferation through TLR4 signaling pathway

**Fig. 7:**
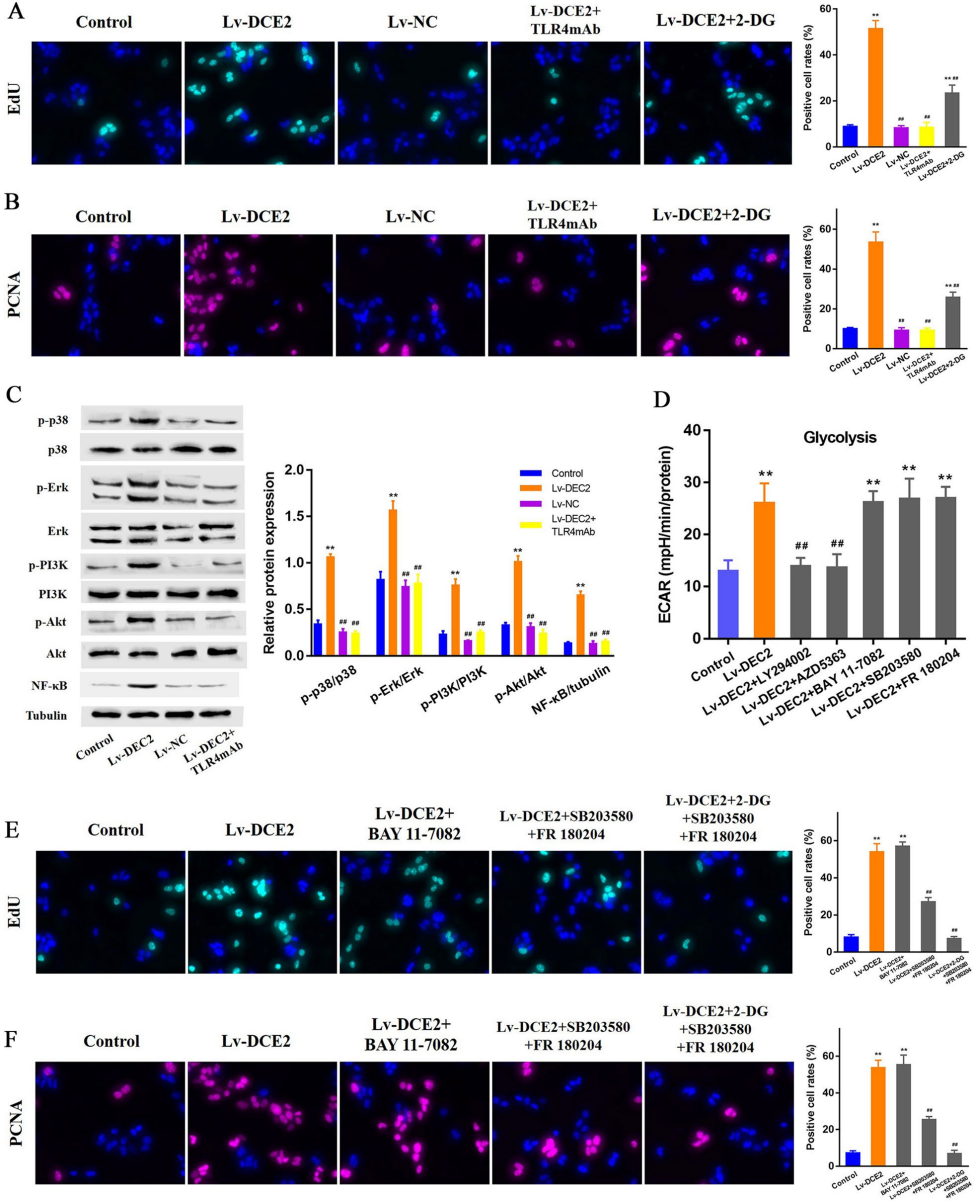
DEC2 regulates MCs proliferation through TLR4 signaling pathway. **A**, **B** Cell proliferation assays indicated that DEC2-induced proliferation could only be partly inhibited by glycolysis inhibitor 2-DG, however, it was completely suppressed after TLR4 inhibitor CP treatment. **C** P38 MAPK, Erk, PI3K/Akt and NF-κB were found activated in MCs with overexpression of DEC2. These pathways were all inhibited after CP treatment. **D** Use of multiple inhibitors showed that PI3K/AKT pathway was associated with DEC2-induced glycolysis activation, and p38 MAPK, ERK and NF-κB were independent of this process. **E**, **F** The cell proliferation assays indicated that suppression of p38 MAPK and ERK signaling can partly inhibited DEC2-induced MCs proliferation, and combined use of p38 MAPK/ERK inhibitors and 2-DG completely inhibited DEC2-induced MCs proliferation. **P < 0.01 vs. control; ^##^*P* < 0.01 vs. Lv-DEC2

To determine whether glycolysis activation is responsible for DEC2-induced MCs proliferation, EdU staining was used to evaluate the proliferation of MCs with overexpression of DEC2 and glycolysis inhibitor 2-DG. Significantly increased MCs proliferation was observed in MCs with overexpression of DEC2. This elevation could be completely suppressed after CP treatment, but only partly inhibited by 2-DG treatment (Fig. 7A). This result hints that other signaling pathway participated in DEC2/TLR4-induced MCs proliferation except for glycolysis. These data were further confirmed by the expression of PCNA in MCs with overexpression of DEC2 and 2-DG (Fig. 7B). Multiple signaling pathway proteins were screened for participating in the DEC2/TLR4-induced MCs proliferation. P38 MAPK, Erk, PI3K/Akt and NF-κB were found activated in MCs with overexpression of DEC2. These pathways were all inhibited after CP treatment (Fig. 7C). To distinguish the signaling pathways associated with or without glycolysis-related MCs proliferation, corresponding inhibitors were used to detect the change of glycolysis level in DEC2-overexpressed MCs. The result showed that PI3K/AKT pathway was associated with DEC2-induced glycolysis activation, and p38 MAPK, ERK and NF-κB were independent of this process (All P < 0.01, Fig. 7D). The cell proliferation assay indicated that inhibition of NF-κB signaling could not alter DEC2-induced MCs proliferation, however, suppression of p38 MAPK and Erk signaling can partly inhibited DEC2-induced MCs proliferation. Furthermore, using p38 MAPK/ERK inhibitors and 2-DG simultaneously completely inhibited DEC2-induced MCs proliferation (Fig. 7E). These data suggest that DEC2 regulates MCs proliferation through two signaling pathways including dependent and independent of glycolysis, which located in the downstream of TLR4 signaling. These data were further confirmed by the staining of PCNA in MCs (Fig. 7F).

## Discussion

LN, an important risk factor for mortality in systemic lupus erythematosus, is one kind of immune complex-mediated glomerulonephritis (Lech and Anders 2013). The pathogenesis of LN is associated with the activation of a cluster of neutrophil-associated genes (Banchereau et al. 2016). Control of inflammation with corticosteroids and inhibition of autoimmunity by mycophenolate mofetil are currently treatment regimens for LN (Almaani et al. 2017). In glomerulus, MCs participate in the process of injury repair and provide structural support for renal tissues (Nowling 2022). Excessive proliferation of MCs is a hallmark for their activation by inflammation or other stimuli during chronic renal disease. Multiple bioactive factors including cytokines, fibrotic factors and growth factors released by MCs aggravate the renal injury and dysfunction in LN. In addition, proliferative MCs compress the glomerular capillaries and result in glomerular ischemia and disorganization. Thus, in this study, we focused on the modulation of excessively proliferative MCs during the development of LN.

Serum from the LN model animals or patients is suitable to simulate the LN circumstance exposed to MCs (Liu et al. 2019). In the current study, the MCs were exposed to the serum from 32-week-old Fcgr2b^−/−^ mice, a LN model proved by proteinuria and pathological changes of renal tissues. Increased expression of DEC2 was found not only in the kidney of Fcgr2b^−/−^ mice, but also in the MCs treated with LN serum, compared to those in corresponding controls. Furthermore, proliferative markers including PCNA expression and EdU staining suggested that excessive proliferation was observed in the kidney of Fcgr2b^−/−^ mice and in the LN serum-treated MCs. To determine whether proliferative MCs are associated with the expression of DEC2, we knocked down the expression of DEC2 both in renal tissue of mice and in MCs. The results indicated that decreased proliferative markers were detected in both renal tissue and MCs exposed to LN circumstance. In addition, alleviated proteinuria and renal tissue compression were found in DEC2-knocked down mice. These data suggest that DEC2 plays critical role in regulating the proliferation of MCs in LN.

Cell proliferation is modulated by variety of proliferative signals from extracellular and intracellular environment. Energy metabolism is also a factor that cannot be ignored to promote the cell proliferation (Lunt and Vander Heiden 2011; Martínez-Reyes and Chandel 2021). The bioenergetics that support this process remain poorly elucidated. In activated cells with proliferative behavior, increased glycolysis and net lactate production are commonly observed even the cells cultured in oxygen rich environment (Beck et al. 2015). Thus, the glucose metabolism was evaluated in LN serum-treated MCs. Several parameters including lactate production, glucose consumption, ATP production and mitochondrial membrane potential were assayed in the MCs. The results showed that LN serum treatment significantly increased the levels of abovementioned parameters, suggesting that cellular energy metabolism is involved in the proliferation of MCs in LN. Furthermore, knockdown of DEC2 partly reversed the increase of abovementioned parameters, indicating that DEC2 is associated with the glucose metabolism in LN serum-treated MCs.

To elucidate the regulative mechanism of bioenergetics in MCs proliferation and the role of DEC2, we analyzed the glycolysis and mitochondrial respiration function in this process. By dynamically monitoring ECAR of the MCs along with the sequential addition of glucose, oligomycin and 2-DG, multiple glycolysis-related parameters including glycolysis, glycolytic capacity and glycolytic reserve can be calculated. Both glycolysis level and glycolytic capacity significantly increased in the LN serum-treated MCs compared with those in controls, suggesting that LN serum activates the glycolysis in MCs. These increases in glycolysis level and glycolytic capacity can be partly reversed after knockdown of DEC2 in the MCs, indicating that DEC2 regulates glycolysis in LN serum-treated MCs. Then we evaluate the mitochondrial respiration function in MCs. By dynamically monitoring OCR of the MCs along with the sequential addition of oligomycin, FCCP and rotenone/antimycin A, multiple mitochondrial respiration-related parameters including basal respiration, ATP production-linked respiration, maximal respiration, spare capacity and proton leak can be calculated. LN serum treatment can not influence the basal respiration and ATP production-linked respiration of the MCs, and no obvious effects can be found in DEC2-knocked down cells. These data hint that energy provided for MCs proliferation seems dose not come from the mitochondrial respiration. However, increased maximal respiration and spare capacity were found in the LN serum-treated MCs compared with those in controls, and DEC2 knockdown partly reversed these changes. Spare capacity is an important index representing mitochondrial fitness, and increased spare capacity is indicative of elevated mitochondrial activity (Everts et al. 2014). Increased spare capacity but not basal respiration in LN serum-treated MCs can be explained by the flux of glycolytic carbon into the tricarboxylic acid cycle. These data suggest that DEC2 is associated with mitochondrial fitness in LN serum-treated MCs. Enhanced glycolysis is also a feature of Warburg effect, which contributes to the proliferation of multiple type of cells including cancer cells. Our findings suggest that increased glycolysis is a metabolic characteristic for activated MCs.

Lastly, we reported that downregulation of DEC2 and the TLR4 signaling inactivation inhibited the proliferation of MCs (Qi et al. 2020). To determine whether DEC2 regulates glycolysis in MCs through TLR4 pathway, we detected the expression of TLR4 in the MCs. Firstly, LN serum activated TLR4 signaling, and DEC2 knockdown inhibited the LN serum-induced TLR4 activation in MCs. Overexpression of DEC2 could also activate the TLR4 signaling. Secondly, overexpression of DEC2 significantly activated the glycolysis and glycolytic capacity in MCs, and TLR4 inhibitor CP effectively suppressed the elevation of ECAR values. In dendritic cells, TLR agonist increases lactate production and activates glycolysis through up-regulation of the expression of GLUT1 (Tannahill and O'Neill 2011; Krawczyk et al. 2010). In this study, we revealed that overexpression of DEC2 increased the expression of GLUT1 in MCs and CP treatment suppressed the elevation of GLUT1 expression. These data indicate that DEC2 activates MCs glycolysis through TLR4 signaling and GLUT1 regulation.

To determine whether glycolysis activation is responsible for DEC2-induced MCs proliferation, we evaluated the proliferation of the MCs after treatment with glycolysis inhibitor 2-DG. Results showed that DEC2-induced proliferation could only be partly inhibited by 2-DG, however, it was completely suppressed after CP treatment. These data hint that other signaling pathway may participate in DEC2/TLR4-induced MCs proliferation except for glycolysis. Then we screened multiple signaling pathway proteins, and found that p38 MAPK, Erk, PI3K/Akt and NF-κB were activated in MCs with overexpression of DEC2. These pathways can be inhibited after CP treatment. To distinguish the signaling pathways associated with or without glycolysis-related MCs proliferation, corresponding inhibitors were used to detect the change of glycolysis level in DEC2-overexpressed MCs. The result showed that PI3K/AKT pathway was associated with DEC2-induced glycolysis activation, and p38 MAPK, ERK and NF-κB were independent of this process. The cell proliferation assay indicated that suppression of p38 MAPK and ERK signaling can partly inhibited DEC2-induced MCs proliferation, and combined use of p38 MAPK/ERK inhibitors and 2-DG completely inhibited DEC2-induced MCs proliferation. These data confirm that DEC2 regulates MCs proliferation through two signaling pathways including dependent and independent of glycolysis, which locates in the downstream of TLR4 signaling.

## Conclusion

In conclusion, to elucidate the mechanism by which DEC2 modulates the proliferation of MCs in LN would provide a better understanding of LN progression, and reveal a potential target for LN treatment. Here we found that DEC2 regulates MCs proliferation through TLR4 signaling pathway, and unexpectedly, we identified two pathways located in the downstream of TLR4 signaling. One is glycolysis-dependent, and modulated by PI3K/AKT pathway. The other one is glycolysis-independent, and controlled by p38 MAPK and Erk signaling. Knockdown of DEC2 expression inhibits the proliferation of MCs through suppressed glycolysis and p38 MAPK/Erk pathway in LN.

## Data Availability

The data and code generated or analyzed in this study are available from the corresponding authors upon reasonable request.
