# Correction: Knockdown of DEC2 expression inhibits the proliferation of mesangial cells through suppressed glycolysis and p38 MAPK/Erk pathway in lupus nephritis

**DOI:** 10.1186/s10020-023-00725-3

**Published:** 2023-09-14

**Authors:** Huimeng Qi, Li Xu, Qiang Liu

**Affiliations:** 1 Department of General Practice, Fuyang Hospital of Anhui Medical University, Fuyang, 236000 Anhui Province China; 2 Department of Clinical Laboratory, The First Affiliated Hospital of China Medical University, Shenyang, 110001 Liaoning Province China; 3 Department of Nephrology, Guangdong Second Provincial General Hospital, Guangzhou, 510317 Guangdong Province China

**Correction: Molecular Medicine (2023) 29:99** 10.1186/s10020-023-00672-z

Following publication of the original article (Qi et al. 2023), the authors informed us that they were incorrectly affiliated.

This Correction article shows the correct affiliations.
